# Silent Thrombotic Thrombocytopenic Purpura: PLASMIC, Lessons Learned, and Current Management Overview

**DOI:** 10.7759/cureus.13803

**Published:** 2021-03-10

**Authors:** Alexandra Pisklakova, Joshua Barbir, Jan-Paul Sambataro, Christian Almanzar, Faiza Manji

**Affiliations:** 1 Internal Medicine, Brandon Regional Hospital, Tampa, USA; 2 Oncology, Brandon Regional Hospital, Tampa, USA

**Keywords:** ttp, thrombocytopenia, hemolytic anemia, plasmic, hemolysis, adamts13

## Abstract

Thrombotic thrombocytopenic purpura (TTP) is a rare, life-threatening autoimmune or hereditary thrombotic microangiopathy (TMA) that may be difficult to recognize given the wide spectrum of presenting symptoms. The clinical diagnosis of TTP is based on thrombocytopenia, microangiopathic hemolytic anemia and is confirmed by a disintegrin-like and metalloproteinase with thrombospondin type one motif, member 13 (ADAMTS13) <10%. However, the latter confirmation is not rapidly available, and treatment is typically initiated based on the degree of clinical suspicion. The PLASMIC score was recently developed to distinguish between TMA patients with and without severe ADAMTS13 deficiency and used as an adjunct in the diagnosis of TTP when the clinical picture is not clear.

Here we present the case of a completely asymptomatic female with no past medical history diagnosed with TTP after evaluation for thrombocytopenia found on a routine wellness visit. A high PLASMIC score was crucial in the decision to initiate treatment given an unusual asymptomatic presentation.

## Introduction

Thrombotic thrombocytopenic purpura (TTP) is a rare hematologic disease with an average annual prevalence of 10 cases/million people and an annual incidence of one new case/million people [1,2]. Thrombotic thrombocytopenic purpura is characterized by widespread formation of microthrombi, rich in von Willebrand factor (vWF) and platelets, which ultimately causes consumptive thrombocytopenia, hemolysis, and impaired tissue perfusion [3]. It is distinguished by an immune-mediated deficiency of a disintegrin-like and metalloproteinase with thrombospondin type one motif, member 13 (ADAMTS13). TTP is mainly caused by an autoimmune mechanism, but rare nonimmune inherited forms, also known as Upshaw-Schulman syndrome, are described where ADAMTS13 deficiency results from biallelic mutations in the ADAMTS13 gene [4].

The definition for TTP has changed over time. Initially, an acute episode of TTP was defined by clinical criteria (visceral ischemic symptoms targeting multiple organ systems) and standard laboratory criteria (microangiopathic hemolytic anemia and severe thrombocytopenia) occurring in the absence of other apparent etiology. This definition was recently completed by the presence of a severe ADAMTS13 deficiency (activity <10%), which is the only marker specific for TTP (100% sensitivity and 99% specificity) [5]. Only 10% of the patients present with what used to be a classic and now obsolete pentad of thrombocytopenia, fever, neurological symptoms, microangiopathic hemolytic anemia, and renal insufficiency [6]. Since the presenting symptoms and their severities can vary significantly, there is a substantial risk of delay in treatment and it might be catastrophic as TTP has a mortality rate exceeding 90% in the absence of appropriate treatment. 

The PLASMIC diagnostic model was recently introduced and validated to assess reliably and distinguish between TMA patients with and without severe ADAMTS13 deficiency (defined as ADAMTS13 activity level ≤10%) and used as an adjunct in the diagnosis of TTP when the clinical picture is not clear [7].

Here we present the case of a completely asymptomatic female with no past medical history diagnosed with TTP after evaluation for thrombocytopenia found on a routine wellness visit. The PLASMIC score was crucial in the decision to initiate treatment given an unusual asymptomatic presentation. 

## Case presentation

A 34-year-old female with no significant past medical history was sent into the hospital ED by a primary care physician for evaluation of low platelet count. On initial assessment, review of symptoms was completely negative. The patient reported no history of abnormal bleeding, easy bruising, or changes in menstruation. She was taking iron and folate supplements during pregnancy three years ago; however, she stopped taking them after delivery. She denied any history of miscarriages and reported only one full-term pregnancy with no complications. The patient denied any recent infection or flu-like symptoms. Her child was in daycare and up to date with vaccinations. Respiratory, cardiovascular, gastrointestinal tract, and neurological examinations were unremarkable.

Laboratory studies (Table 1) revealed macrocytic hypochromic anemia (hemoglobin 9.5 g/dL, mean corpuscular volume [MCV] 97.9 fL) with reticulocyte count of 7.2% (immature reticulocyte fraction 27.8%), and thrombocytopenia (platelet count 20,000/cubic millimeter). Lactate dehydrogenase (LDH) was 489 U/L (reference range: 100 - 250 U/L) and haptoglobin was undetectable. Prothrombin time, partial thromboplastin time, renal and hepatobiliary function were within normal limits. Serology for hepatitis, human immunodeficiency virus (HIV), Epstein-Barr virus (EBV), cytomegalovirus (CMV), parvovirus and direct Coombs tests were negative. A review of peripheral smear on admission was remarkable for markedly decreased platelet count, large platelets and normal red blood cell (RBC) morphology and no schistocytes. The PLASMIC score was calculated (Table 2) and found to be 6, which prompted the ADAMTS13 level check.

**Table 1:** Patient's laboratory test results upon admission INR: international normalized ratio, BUN: blood urea nitrogen, AST: aspartate aminotransferase, ALT: alanine aminotransferase, ESR: erythrocyte sedimentation rate, ANA: antinuclear antibody, CMV: Cytomegalovirus, EBV: Epstein-Barr virus, HAV: hepatitis A virus, HBV: hepatitis B virus, HCV: hepatitis C virus, SARS-CoV-2: severe acute respiratory syndrome coronavirus 2, TSH: thyroid stimulating hormone, Ig: immunoglobulin, PCR: polymerase chain reaction

Laboratory values	Reference Range	Day 1	Day 10
Haptoglobin	30 - 200 mg/dL	< 20	86.6
Reticulocyte count	0.5 - 1.7 %	7.2	11.44
Fibrinogen	200 - 500 mg/dL	372	
INR	0.9 - 1.1	1	1
Creatinine	0.55 - 1 mg/dL	0.86	0.91
BUN	7 -18 mg/dL	13	18
Total Bilirubin	0.2 - 1 mg/dL	1	0.6
AST	15 - 37 Units/L	22	33
ALT	13 - 56 Units/L	16	54
C-reactive protein	0 - 0.3 mg/dL	0.5	
ESR Westergren	0 - 20 mm/hr		
ANA	Negative	Negative	
Complement C3	90 - 180 mg/dL	86	
Complement C4	14 - 44 mg/dL	16.5	
ADAMST-13 Inhibitor	< 0.4 IU	< 0.4	
CMV DNA PCR	Not detected	Not detected	
EBV IgG	0 - 0.8 AI	>8	
EBV IgM	Not detected	Not detected	
HAV IgM Ab	Not detected	Not detected	
HBV Antigen	Not detected	Not detected	
HCV Antibody	Not detected	Not detected	
Monoscreen	Negative	Negative	
HIV p24 and HIV Ab	Not detected	Not detected	
Parvovirus B19 DNA PCR	Not detected	Not detected	
SARS-CoV-2 Ag	Not detected	Not detected	
Copper	72 - 166 ug/dL	119	
Vitamin B12	211 - 911 pg/mL	616	
Folate	> 2.76 ng/mL	12.6	
TSH	0.36 - 3.74 uIU/mL	1.75	
Ferritin	8 - 388 ng/mL	600	
Iron	35 - 150 ug/dL	62	

**Table 2:** PLASMIC score criteria "relative to patient outcomes" PLASMIC score composed of the following criteria: a platelet count < 30,000/μL, evidence of hemolysis (reticulocyte count > 2.5%, elevated indirect bilirubin > 2 mg/dL, undetectable to low haptoglobin levels), mean corpuscular volume < 90 fl, international normalized ratio < 1.5, and creatinine < 2 mg/dl with no active cancer or organ/stem cell transplant. One point is given to each of the above met criteria. Score of 0–4 suggests low risk for TTP (0 - 4%).  Score of 5 suggests intermediate risk for TTP (5 - 25%) and plasma exchange initiation should be considered. Score of 6 and above suggests high risk for TTP (60 - 80%) and plasma exchange should be immediate if clinical suspicion is high. MCV: mean corpuscular volume, INR: international normalized ratio

	Parameter	Points	Our Patient
P	Platelet count < 30 x 109/L	1	1
L	Combined hemolysis parameter: Indirect bilirubin > 2mg/dL, OR Reticulocyte count > 2.5%, OR Haptoglobin undetectable	1	1
A	No active cancer	1	1
S	No history of solid organ or stem cell transplant	1	1
M	MCV < 90 fL	1	0
I	INR < 1.5	1	1
C	Creatinine < 2.0 mg/dL	1	1

**Figure 1:**
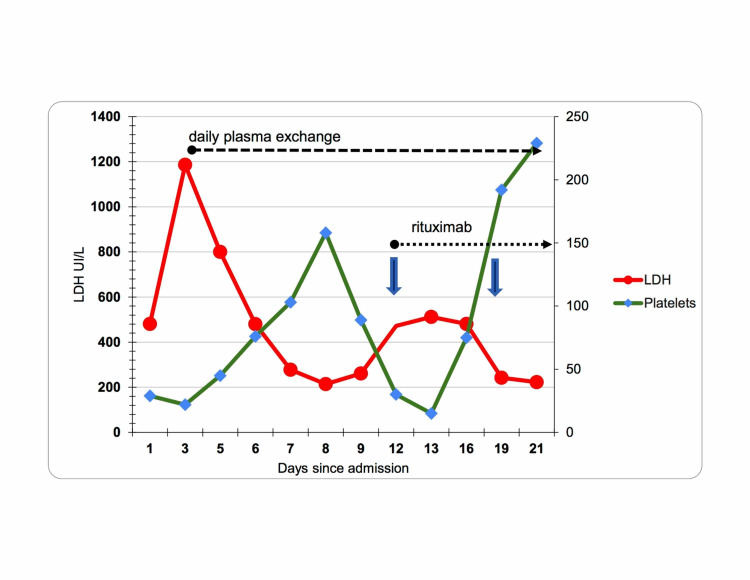
Graphic representation of the patient's laboratory test values (platelets and LDH) trends before and during treatment LDH: lactate dehydrogenase

The following day, laboratory studies revealed stable blood count and a significant increase in LDH to 1186 U/L (reference range: 100-250 U/L). The patient remained completely asymptomatic. A review of peripheral smear was remarkable for three to four schistocytes per high-power field. Based on the PLASMIC score of 6, diagnosis of TTP was highly favored and therapy with daily plasma exchange (PEX) along with prednisone, 80 mg (1 mg/kg) was initiated. ADAMTS13 activity was <2% confirming the diagnosis of TTP. After one week of PEX, the platelet count increased but remained in a 50,000/cubic millimeter range. LDH initially normalized; however, by day 11, LDH increased to 409 U/L (Figure 1). ADAMTS13 level remained <2%. Infusion with rituximab 375mg/m2 IV weekly was added to the treatment regimen as refractory TTP was suspected. The patient had a good response with a platelet count remaining over 150,000/cubic millimeter for two days. PEX was discontinued after 14 days of daily treatments. 

In the outpatient setting, the patient continued to receive weekly rituximab infusions and oral prednisone taper and progressed well with evidence of recovery, as demonstrated by normalized platelet count, LDH, and reticulocytes. She completed four weeks of rituximab with no evidence of recurrence.

On a last note, evaluation for the underlying cause of TTP included genetic testing to rule out congenital TTP which revealed no compound heterozygous state with mutations in exons 24 and 13 of the ADAMTS13 locus. Screening for autoimmune and other hematological diseases, which included antinuclear antibody (ANA), serum complement levels, flow cytometry, and serum immunoglobulin levels, was negative as well.

## Discussion

TTP is a true medical emergency and rapid recognition is crucial to initiate appropriate treatment. Untreated TTP follows a fulminant course of progressive severe hemolytic anemia, profound thrombocytopenia, neurologic deterioration, cardiac ischemia, irreversible renal damage and, ultimately, death [8].

Plasma exchange to supplement ADAMTS13 and removal of ultra-large VWF multimers remains the backbone of treatment and should be initiated as soon as diagnosis of TTP is suspected. It is performed daily (1.0 × patient plasma volume exchange) until platelet count has stably recovered and hemolysis has ceased. Historically, high-dose steroids (1 mg/kg/day) have been used together with plasma exchange; however, there is no proven benefit in treatment of TTP based on a number of studies and retrospective reviews [9]. In our case, we used prednisone at the mentioned above dose as a part of pre-medication regimen prior to PEX. 

In 1997, rituximab (humanized anti-CD20 monoclonal antibody) was approved by the US Food and Drug Administration for the treatment of non-Hodgkin lymphomas. Although rituximab is not approved for treatment of TTP, it has been used off-label with increasing frequency since 2002 [10]. The ultimate role of rituximab in the management of patients with TTP remains uncertain, but it is frequently used in TTP with suboptimal response to PEX, refractory and recurrent TTP. The optimal dose and schedule of rituximab therapy for TTP is still not established, however the most frequently used dose is 375 mg/m2 once weekly for four weeks [10]. Given the high efficacy, a number of studies debated whether rituximab should be the frontline of treatment; nonetheless no consensus has been reached as of today [11]. In our case, we opted for rituximab treatment after poor response to one week of standard therapy with PEX. The patient responded to rituximab in five days (Figure 1) based on a stable platelet count and decreasing LDH and reticulocyte count. Rituximab was administered immediately after PEX with the next session of PEX in 24 hours to ensure the drug is not removed from the bloodstream.

Cyclosporine, cyclophosphamide, and vincristine have historically been used in refractory TTP and are now considered salvage therapy when all else fails. Splenectomy is also a known treatment but it remains controversial. Quite frankly the benefit of splenectomy is uncertain, especially considering post-splenectomy complications. 

In 2019, a novel anti‐VWF humanized single-variable domain nanobody, caplacizumab, was approved by FDA for treatment of TTP. Caplacizumab, in addition to conventional treatment, led to a rapid recovery in comparison with conventional treatment plus placebo, without increasing significant hemorrhagic complications [12]. Safety profile and efficacy are encouraging; regardless, future studies are needed to guide the duration of therapy and the potential role of caplacizumab in refractory and relapsed TTP.

## Conclusions

TTP is an exceedingly rare life-threatening hematologic disease; thus, appropriate diagnosis in a medical emergency is challenging to recognize given the wide spectrum of presenting symptoms. A delay in treatment may be catastrophic and a high index of suspicion is decisive. The ease of calculating the PLASMIC score makes it a valuable clinical tool when ADAMTS13 testing is not available, the clinical picture is not well defined, and an urgent decision whether to initiate the PEX treatment is needed. In this report, we emphasize the crucial role of the PLASMIC score in the decision to initiate treatment given an unusual asymptomatic presentation.
